# From the Analysis of Anatomy and Locomotor Function of Biological Foot Systems to the Design of Bionic Foot: An Example of the Webbed Foot of the Mallard

**DOI:** 10.3390/biomimetics8080592

**Published:** 2023-12-05

**Authors:** Dianlei Han, Hairui Liu, Lizhi Ren, Jinrui Hu, Qizhi Yang

**Affiliations:** School of Agricultural Engineering, Jiangsu University, Zhenjiang 212013, China; 2212216053@stmail.ujs.edu.cn (H.L.); 2212316050@stmail.ujs.edu.cn (L.R.); 2222316034@stmail.ujs.edu.cn (J.H.); yangqz@ujs.edu.cn (Q.Y.)

**Keywords:** mallard, webbed foot, osteomuscular system, gross anatomy, CT, motor biomechanical function

## Abstract

This study utilized the mallard’s foot as the subject, examining the bone distribution via computed tomography (CT) and analyzing pertinent parameters of the tarsometatarsal bones. Additionally, gross anatomy methods were employed to elucidate the characteristics of the toes and webbing bio-structures and their material composition. Biologically, the mallard’s foot comprises tarsometatarsal bones and 10 phalanges, enveloped by fascia, tendons, and skin. Vernier calipers were used to measure the bones, followed by statistical analysis to acquire structural data. Tendons, originating in proximal muscles and terminating in distal bones beneath the fascia, facilitate force transmission and systematic movement of each segment’s bones. Regarding material composition, the skin layer serves both encapsulation and wrapping functions. Fat pads, located on the metatarsal side of metatarsophalangeal joints and each phalanx, function as cushioning shock absorbers. The correlation between the force applied to the tarsometatarsal bones and the webbing opening angle was explored using a texture analyzer. A simplified model describing the driving force behind the webbing opening angle was introduced. Furthermore, we designed a bionic foot, contributing a foundational reference for anti-sinking bionic foot development.

## 1. Introduction

Mallards (*Anas platyrhynchos*) reside in various terrains, such as sandbars and reservoirs, throughout the year, with their survival in these soft-ground environments intricately linked to their anatomical structure. As bipedal avians, mallards’ unique toe configuration facilitates their mobility on yielding terrain. They possess four toes, of which the Ⅰ toe has undergone evolutionary diminution, rendering the Ⅱ, Ⅲ, and Ⅳ toes crucial for ambulation and bodily support. Additionally, their webbed feet serve pivotal roles in stabilizing sand, minimizing displacement, and augmenting the surface contact area, thereby optimizing movement on soft substrates like mudflats.

Benjamin et al. [1] explored tendon functionality and its nutritional sustenance from tendon vasculature, emphasizing the critical interplay between tendons and fasciae. They highlighted how robust muscles contribute to tendons, ensuring the limb’s distal extremity remains unencumbered by mass and preserving its functional integrity. Biewener [2] undertook an anatomical appraisal of equines, juxtaposing tendon elastic energy conservation with mechanical work. His findings revealed that elastic energy recovery accounted for approximately 40% of the mechanical effort during transitions from walking to a moderate trot in horses. This phenomenon underscores the specialized constitution of elongated tendons relative to diminutive pinnate muscle fibers, indicative of an evolutionary adaptation for efficient muscular force application and enhanced elastic energy recapture. Gangl et al. [3] provided an exhaustive anatomical account of ostrich hind limbs, detailing the musculature and tendons affiliated with various skeletal components and discussing the structural principles underpinning the entire hind limb. Their insights particularly pertained to musculotendinous architectures instrumental in energy-efficient bipedal locomotion. Schaller et al. [4] delved into the biomechanics of the ostrich’s intertarsal joint. Their research illuminated the presence of two critical ligaments at the junction, functioning akin to cords that facilitate the joint’s repositioning during extension or flexion. This study elucidated the correlation between intertarsal joint angular alterations and the requisite joint torque, laying the foundational groundwork for bionic flexible tensor-tendon joint design. Zhang et al. [5] conducted an extensive anatomical analysis of the ostrich’s foot locomotor apparatus. Their investigation thoroughly examined the skeletal, tendinous, and ligamentous constituents, discovering that dual interphalangeal ligaments regulate the fourth toe’s movement, predominantly influenced by the lateral interphalangeal ligament, with negligible contribution from the internal counterpart. Clifton et al. [6] evaluated the hindlimb musculature and webbing’s propulsive capabilities in aquatic avian species, noting that adept divers streamline their bodies by adjoining the femur and tibia to the thorax, concealed beneath the abdominal skin. They found that the lateral calf flexor undergoes near-isometric contraction during swimming, suggesting its role in averting hip and knee movements throughout the power stroke, thereby conserving a streamlined form. These studies collectively enhance our understanding of tendon and bone distribution via comprehensive anatomical exploration.

Weissengruber et al. [7] investigated elephant footpads and found that the plantar configuration, which is mostly made up of connective tissue strips and adipocytes, plays a big role in how force is distributed and how mechanical energy is absorbed or stored during weight-bearing activities. EI-Gendy et al. [8] dissected ostrich feet to discern structural-functional adaptations conducive to long-distance travel. Their analysis of the footpads, integral to ground contact, revealed four distinct pads: two on the third toe, one on the fourth, and one at the metatarsophalangeal joint. The study detailed papillae structures on the pads’ ventral surface and the longitudinal skeletal-cartilaginous formations beneath the phalanges, highlighting the soft tissues’ intricate arrangement. Different papillae orientations, lengths, and thicknesses were seen under electron microscopy. The collagen fibers’ parallel or slanted alignments in the pads may help ostriches absorb shock and run for long periods of time. Miao et al. [9] explored the impact attenuation properties of German Shepherd Dogs’ footpads. They found the pad to have a complex, tripartite structure: the epithelium, dermis, and subcutis. The epithelium significantly mitigates ground impact forces, whereas the dermis and subcutis function as a hydrostatic system for energy storage, release, and dissipation, collaboratively catering to the biomechanical requisites of locomotion. Zhang et al. [10] examined the ostrich footpad’s cushioning and vibration-damping capabilities, biomechanical testing, and finite element analysis. They determined that the footpad, comprising sequential skin, fascia, and toe cushion layers, diminishes stress from the exterior inward. With a minimum response frequency of 164.22 Hz, the footpad adeptly avoids resonance, significantly reducing peak acceleration through its composite material construction. Tian et al. [11] analyzed the shock-absorbing mechanisms of goat hoof ball tissues and identified the primary components as the epidermis, dermis, dermal papillae, and subcutaneous tissue, with the dermal papillae dispersed within the dermis. Stress concentrations in the epidermis and uniform distribution in the dermis and dermal papillae suggest the epidermis’ role in diminishing ground impact forces, whereas the dermis facilitates energy storage, release, and dissipation, all collaboratively contributing to the hoof ball tissues’ cushioning effect. Collectively, these studies elucidate the footpads’ essential functions in shock absorption or energy conservation during animal locomotion.

Zhang et al. [12] constructed a three-dimensional ostrich foot model. They acquired image data via CT scans and segmented these images with medical imaging and Mimics editing software (version 14.0). Geomagic Studio software (version 12.0) facilitated the geometric reconstruction of pertinent tissues, establishing a comprehensive three-dimensional model. In this process, bones, cartilage, and soft tissues that are important to the biomechanics of the ostrich foot were taken out, put back together, and extracted again. This made it possible to study the foot’s high-speed, heavy-duty, and shock-absorbing mechanisms in more detail. Rankin et al. [13] explored the functional contributions of limb muscles and tendons to ostrich locomotion. Their findings indicated that muscle functions in support, propulsion, and braking were contingent on muscle type, location, and the bird’s gait. Additionally, the tendons’ connective and energy-storing roles could be replicated by spring-like structures, informing the design of bionic limbs and feet. Harrison et al. [14] analyzed muscle forces, joint contact pressures, and the storage and use of elastic strain energy in horses’ distal forelimbs, proposing a simplified tendon-driven model. They found that the long flexor tendons in the forelimb’s distal region could store and utilize significant elastic strain energy, particularly while bearing weight. These studies collectively enhanced the understanding of the foot’s assembled structure and the reconstructed foot mechanism, with CT findings offering profound insights.

Lafortune et al. [15] used an accelerometer-equipped force platform mounted on the wall to measure the shocks and external impact forces that the human calf could handle. This led to the idea of a human-ground impact mechanics model. In a distinct study, Taylor-Burt and Biewener [16] investigated the kinematics of mallard ducks’ hind-limbs and the operational dynamics of crucial propulsive muscles during vertical takeoffs on land and in water. They discovered alterations in hindlimb kinematics and muscle functionality, with a notable surge in muscle power output during aquatic takeoffs. Expanding this research, Han et al. [17] scrutinized the kinematic configurations of the mallard’s webbed foot. Their findings highlighted that mallards favor walking or grounded gaits at velocities under 5 km/h. The mallard’s toes moved in such a way that their distal ends made contact with the ground first, then their proximal ends swiftly followed, and their movements reversed when they lifted off. Intriguingly, the webbing exhibited a synchronized closing before lift-off and reopening before touch-down. This coordination suggests that mallard toes II, III, and IV operate as a cohesive system, with both the toes and webbing contributing collaboratively to terrestrial locomotion. Further, Han et al. [18] observed mallards’ adaptive strategies during locomotion on soft terrain. They noted significant posture adjustments and a reduced touch-down angle, enabling the birds to navigate sinking challenges. Specifically, on sandy surfaces, the birds’ footfalls created depressions that facilitated sand stabilization and minimized displacement. Throughout this process, the mallards’ weight distribution across the extensive webbed area ensured minimal ground pressure, preventing significant sinking during sandy traverses. These behavioral locomotion studies underscore the importance of delving into the underlying mechanisms steering these complex, adaptive movements.

The prosthesis developed by Park et al. [19] comprises two degrees of freedom at the knee and ankle joints. The knee joint’s movement is replicated using a DC motor, and a spring system is employed at the ankle to generate torque and control flexion angles. Additionally, they conducted topology and shape optimization of the foot and lower limb structures to attain an optimal shape and reduce weight. Sun et al. [20] conducted an optimization of the leg and analyzed the impact of various design parameters on the performance of the optimized leg. They implemented this design on a quadruped robot and evaluated its motion performance. Furthermore, they developed a multi-objective topology optimization method that takes into account both the standing and walking positions of the flexible legs.

Current research on mallards predominantly focuses on the morphological and locomotive aspects of their webbed feet, with a noticeable paucity of investigations into the biological structure and material assembly mechanisms underlying the subsidence resistance of these webbed feet. The mallard’s adeptness at maneuvering through soft terrain is intricately tied to its unique foot structure and the synergistic assembly of rigid and pliable materials. This study aims to delve into the biological and material assembly characteristics of mallard webbed feet by acquiring pertinent biological structural parameters, such as those related to toes, bones, and joints, as well as the interconnected assembly of skin, fascia, tendons, and ligaments, employing CT scanning and gross anatomical methodologies. Utilizing a texture analyzer, we examined the correlation between the webbing opening angle and tarsometatarsal pressure, elucidated the biomechanical functions of the mallard’s webbed foot, and subsequently explored its subsidence resistance mechanisms. Drawing insights from the anatomical and kinematic analyses of the mallard, our objective extends to designing a bionic foot that replicates the functional properties of the mallard’s webbed foot.

## 2. Materials and Methods

### 2.1. Micro-CT

For medical imaging data acquisition, this study employed a Bruker SkyScan 1276 micro-CT scanner (Bruker, Leipzig, Germany), renowned for its optimal blend of high resolution, expansive image size, versatile circular and helical scanning capabilities, and efficient low-dose imaging. The CT scanning procedure was tomographic, designed to yield a comprehensive series of distinct cross-sectional image data of the mallard’s foot upon the scan’s completion.

#### 2.1.1. Micro-CT Scan Procedure

The micro-CT scanning commenced subsequent to the stabilization of the mallard’s foot. The established scanning parameters included a ray tube current of 200 uA, voltage settings at 70 kV, layer thickness precisely at 0.25 mm, scanning resolution at 20.329238 μm, exposure duration of 406 ms, and a scanning angular range of 180 degrees. The pixel matrix was set at 3000 × 1080, with the scanning orientation aligning axially with the mallard’s foot. Calibration was consistently maintained by subjecting the body model to identical scanning conditions. Upon the procedure’s completion, a compilation of 8982 raw images was meticulously acquired, ready for subsequent analysis.

#### 2.1.2. Micro-CT Reconstruction

Utilizing the 3D reconstruction software NRecon (version V1.7.4.2, Bruker, Leipzig, Germany), specific areas within the original image set were targeted for reconstruction. Prior to initiating the reconstruction process, a preview of the image to be reconstructed was examined. To minimize image artifacts and ensure optimal reconstruction quality, several parameters were meticulously adjusted: smoothing was set at 5, beam-hardening at 8, and ring artifact correction was applied at 25%, among other fine-tuned settings. Upon finalizing these parameters, the reconstruction commenced by selecting the designated folder, triggering the image reconstruction process.

#### 2.1.3. Micro-CT Analysis

Analysis of the tarsometatarsal region was conducted using the CT Analyzer software (version 1.20.3.0, Bruker, Leipzig, Germany). Consistent parameters were established to facilitate the software’s calculation of various metrics, including tissue volume, bone volume, percent bone volume, bone surface, trabecular number, and trabecular thickness.

### 2.2. Gross Anatomy of Mallard’s Webbed Feet

#### 2.2.1. Cutting and Peeling the Skin

**Figure 1:**
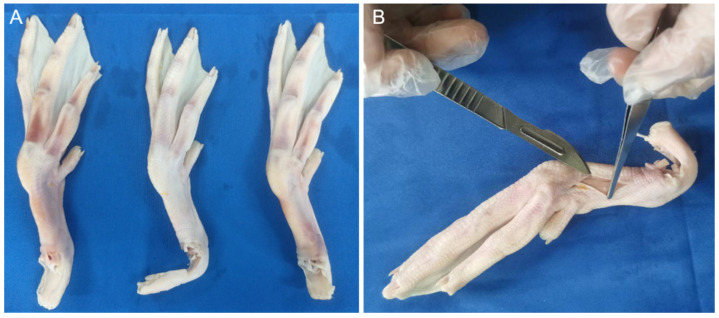
Mallard foot skin peeling process. Anatomical sample (**A**) and skin peel diagram (**B**).

This study employed the feet of adult, healthy mallards, specifically sourced from Shaoxing, Zhejiang Province, China, ensuring no pre-existing foot diseases. To mitigate variances attributable to individual differences, the left foot from three distinct mallards and the right foot from another were dissected (Figure 1A). These specimens were thawed at 2 °C and positioned on a dissection table. Utilizing a scalpel in the right hand and forceps in the left, an incision was initiated near the middle of the tarsometatarsal bone, extending to its distal end. The skin was then punctured perpendicularly at the incision’s start, maintaining a 45-degree angle between the scalpel and skin, facilitating a precise cut. Subsequent to the skin incision, forceps were employed to grip the skin at the incision, pulling it taut. The scalpel was inserted between the skin and fascia, proceeding to carefully separate the skin from the underlying layer (Figure 1B).

#### 2.2.2. Stripping Plantar Toe Pads

Mallards possess pronounced toe pads located beneath the second, third, and fourth toes. Throughout the dissection, each toe pad was methodically excised. An incision was made longitudinally along the toe’s outer skin using a scalpel, after which the skin was delicately elevated with forceps. The scalpel, held adjacent to the tendon sheath, initiated a deeper cut from the proximal position, traversing from the toe’s outer side to the inner and advancing from the base towards the toe tip, thereby detaching the toe pad.

#### 2.2.3. Fascial Layers and Tendon Removal

Upon skin removal, the underlying fascia becomes visible, enveloping tendons and bones. The precise dissection of these fascial layers is crucial for isolating specific biological structures, such as bones and tendons. Given the fascia’s intricate attachment to the tendons, its removal necessitates meticulous attention. Utilizing a scalpel, longitudinal incisions are made along the tissue beneath the fascia, prioritizing complete fascial removal while preserving the integrity of adjacent tissues.

Following the fascia’s removal, the tendons and phalanges are exposed. The tendons connected to the phalanges are carefully detached using the scalpel’s tip, enhancing the visibility of the underlying phalanges.

#### 2.2.4. Measurement and Analysis of Skeletal Parameters

After tendon examination, a more detailed observation of the tarsometatarsal bones and phalanges is conducted. The periosteum, located between the tendons and phalanges, is severed using a scalpel and scissors, freeing it from the bones. Once the bones are isolated, each phalanx is laid out on the dissection table according to its anatomical position for further analysis. To accurately compile comprehensive plantar data on the mallards and derive precise dimensional parameters for subsequent bionic foot research, measurements are taken using vernier calipers. Each tarsometatarsal bone and segment of the phalanges is meticulously measured, with each position undergoing three separate measurements to calculate an average value. These measurements, precise to two decimal points, facilitate a detailed analysis encompassing various structural data. These data include each phalanx’s length, the diameters of the distal phalanx, and measurements at 1/4, 1/2, and 3/4 along the body of the phalanx and the proximal phalanx. The results, expressed as averages, undergo statistical analysis to ensure accuracy and relevance.

### 2.3. Texture Analyzer Test

#### 2.3.1. Test Procedure

**Figure 2:**
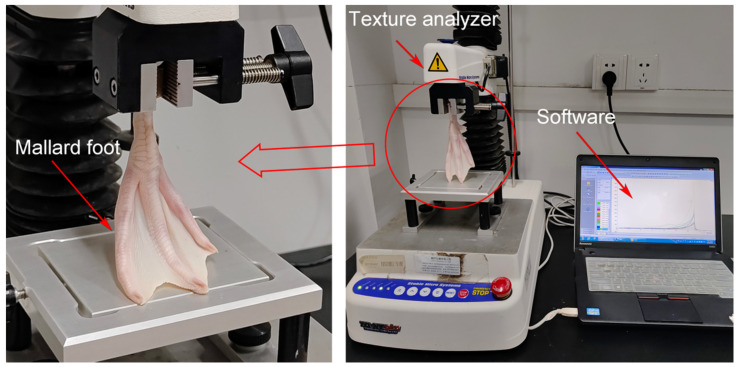
Experimental setup for the texture analyzer.

We utilized the compression mode on a texture analyzer (TA.XTplus, Stable Micro Systems, Godalming, UK) to assess the biomechanical properties of mallard feet. For consistency and to negate individual anomalies, four feet (two left and two right) were isolated for analysis, with each foot undergoing three valid trials. The tarsometatarsal bone’s proximal region was secured within the texture analyzer’s fixture, ensuring a vertical alignment between the bone’s axial direction and the platform (Figure 2). A high-speed camera positioned opposite the spectrometer documented the fluctuations in the foot webbing’s opening angle during testing. The texture analyzer parameters were meticulously set: a testing speed of 0.2 mm/s, preceded by an initial contact speed of 0.1 mm/s. Given the uniformity in foot size, the analyzer was configured to achieve maximum displacement before retracting, with a preset downward shift of 65 mm. Following these settings, the texture analyzer initiated the downward pressure assessment.

#### 2.3.2. Data Processing

At the finishing phase of the test, we procured 12 sets of pressure-time relational data. Concurrently, high-speed camera footage was processed using i-SPEED Suite software (version 2.0, iX Cameras, Rochford, UK), yielding 12 sets of corresponding data reflecting the temporal progression of the toe webbing’s opening angle. This meticulous approach facilitated the generation of pressure-webbing angle relationship graphs, utilizing Origin Pro 2022 (OriginLab Corporation, Northampton, MA, USA) for precise visualization and analysis. These graphical representations elucidated the intrinsic connection between applied pressure and the responsive adjustment in the webbing’s angle. The comprehensive data served as a foundational element in constructing a biomechanical model representative of the test dynamics, thereby providing critical insights into the functional biomechanics underpinning mallard foot movement and pressure response.

## 3. Results

### 3.1. Micro-CT Results

**Figure 3:**
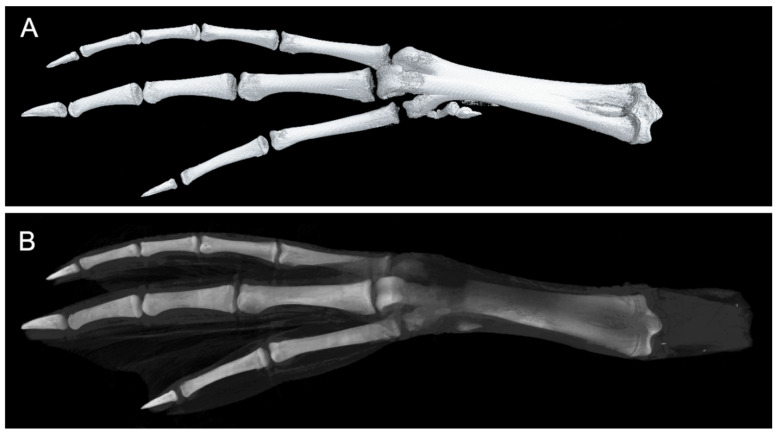
Micro-CT scan results skeletal view (**A**) alongside a skeletal-tissue composite view (**B**), highlighting the translucent nature of the tissue.

The micro-CT scan delineated the intricate structures within the mallard’s foot, categorizing the first, second, third, and fourth toes. Each toe comprised distinct phalanges—numbered 1 through 4—and the associated tissues enveloping them (Figure 3). Notably, the metatarsophalangeal region was articulated into three specific joints: metatarsophalangeal joints II, III, and IV. Each toe culminated in a distinct calcaneal phalanx, readily identifiable in the CT imagery.

In-depth analysis of the tarsometatarsal bones revealed a relatively low bone density, measuring a mere 0.30 g/cm^3^ (Table 1). Despite this, the terminal regions of these bones showcased densely packed trabeculae, exhibiting an average trabecular number of 1.29 1/mm, indicative of robust axial load-bearing capabilities. The Structural Modeling Index (SMI)—a critical parameter in assessing the balance between lamellar (plate-like) and rod structures within the trabecular matrix—provides insights into potential osteoporotic degradation. An SMI nearing 0 suggests a predominance of plate structures, whereas a value closer to 3 indicates an abundance of rod structures. The observed SMI of 0.75 underscores a composition favoring plate structures over rod configurations within the tarsometatarsus.

**Figure 4:**
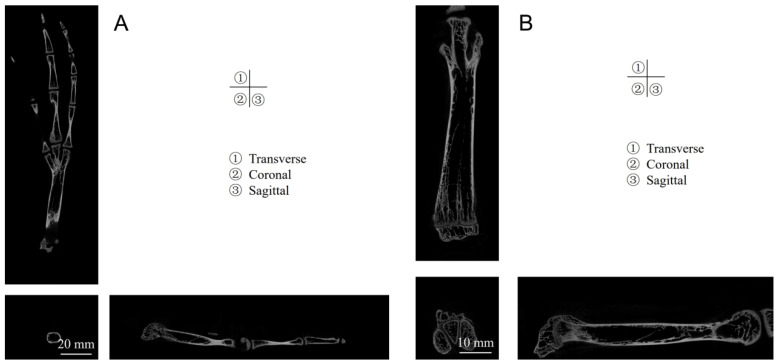
Various micro-CT perspectives: transverse, coronal, and sagittal planes of the foot (**A**) juxtaposed with detailed tarsometatarsal imagery (**B**).

The findings from Figure 4 reveal that both the tarsometatarsal and phalanges possess hollow structures. In the image, white areas represent dense bone with a dense texture, whereas the black areas depict hollow spaces filled with bone marrow, which nourishes the bone. This hollow design aids in reducing the bone’s weight.

### 3.2. Anatomical Observations

#### 3.2.1. Skin, Toe Pads, and Fascia

**Figure 5:**
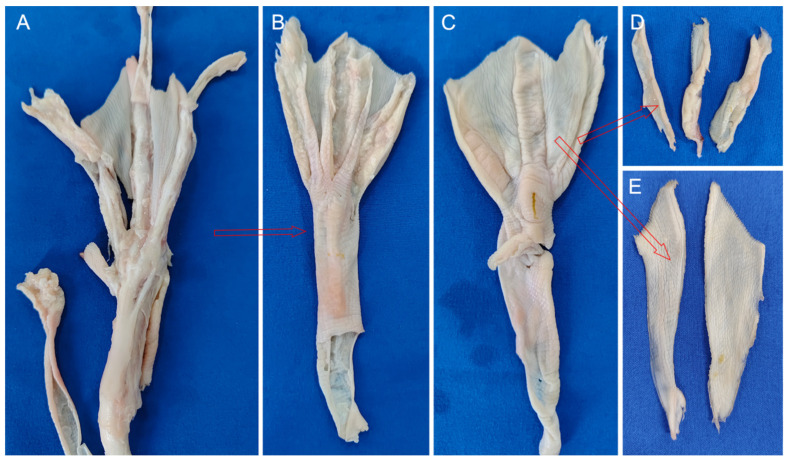
Peeling off the skin layers. The process of peeling off the skin (**A**). The dorsal (**B**) and plantar (**C**) sides of the peeled-off skin. The peeled-off footpads (**D**) and webbing (**E**).

The anatomical exploration of the mallard’s foot reveals intricate structural nuances vital for its locomotive efficiency. Figure 5 illustrates the extracted skin layers, distinctly showcasing the dorsal (B) and plantar (C) aspects, along with the excised webbing (E). This external sheath plays a dual role: it forms a protective barrier for the skeletal components and facilitates locomotion by minimizing environmental resistance. Notably, the toe’s epidermal layer exhibits a series of compact folds, a strategic adaptation to reduce soil adherence during terrestrial movement. Positioned between the second and third toes, as well as the third and fourth toes, the webbing is a marvel of biological engineering. Its delicate, lightweight, and pliable structure enhances locomotive versatility, allowing for expansive stretch during aquatic maneuvers while ensuring minimal soil resistance and effortless detachment when it is off the ground.

Beneath the metatarsophalangeal joints, the plantar region is cushioned by substantial, resilient fat pads (Figure 5D). These pads, predominantly under the proximal segment of the first phalanges of the second, third, and fourth toes, work synergistically with the adjacent ligaments, providing shock absorption and weight distribution upon touching the ground.

**Figure 6:**
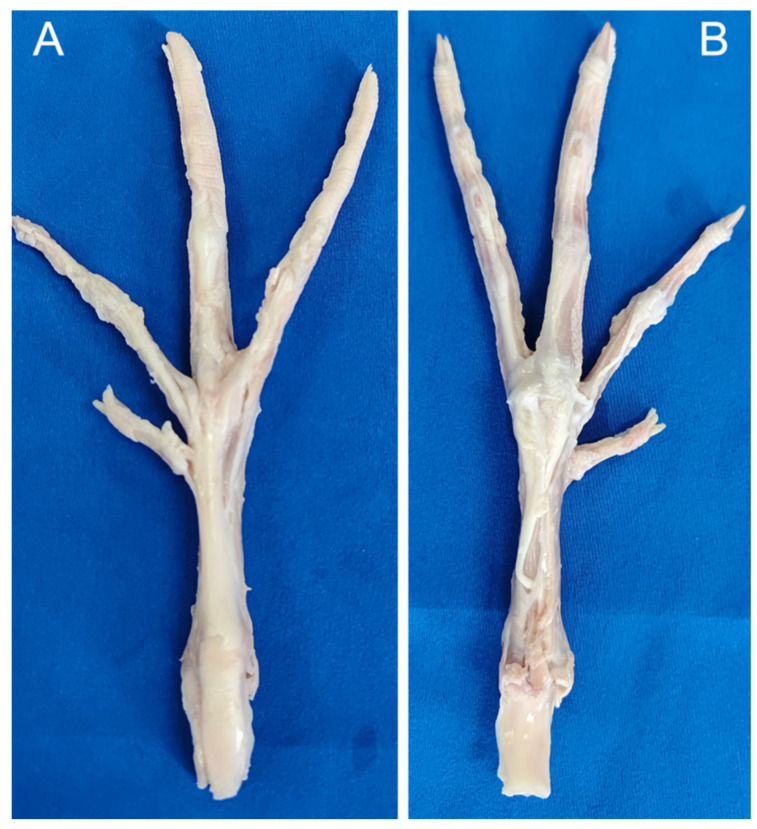
Plantar (**A**) and dorsal (**B**) sides of the foot following the skin’s removal.

After the sequential removal of the skin layer and the fat foot pad, the fascial layer was revealed (Figure 6). The fascial layer is attached to the tendons and bones, and the fascial layer serves as a link and tightener.

#### 3.2.2. Tendons and Ligaments

Tendons, robust bands of fibrous connective tissue, bridge muscles to bones, withstand tension, and facilitate movement through their collagenous composition. The functional dichotomy is evident: plantar flexor tendons govern toe flexion, whereas dorsal extensor tendons oversee their extension.

**Figure 7:**
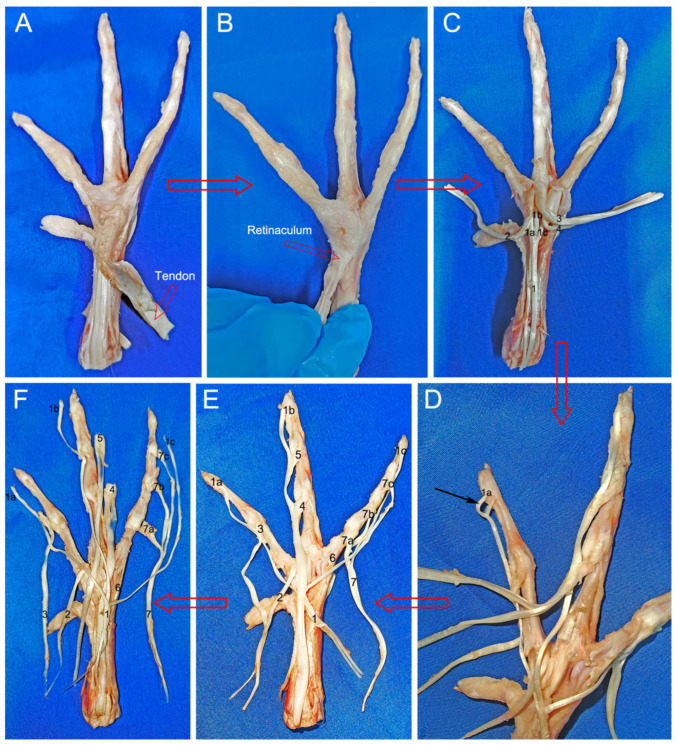
Flexor tendons. The intertarsal joint’s flexor tendon (**A**). The first toe’s retinaculum is connected to the metatarsophalangeal joint retinaculum (**B**). Proximally separated flexor tendon (**C**). The flexor digitorum longus pedis tendon bifurcating distally (**D**). The flexor tendon’s distribution following intertendinous tissue incision (**E**). Cutting at the tendon’s termination point (**F**). 1. Toe II, III, and IV common flexor tendon; 1a. Toe II termination point; 1b. Toe III termination point; 1c. Toe IV termination point; 2. Toe II first flexor tendon; 3. Toe II second flexor tendon; 4. Toe III first flexor tendon; 5. Toe III second flexor tendon; 6. Toe IV first flexor tendon; 7. Toe IV second flexor tendon; 7a. Toe IV termination point; 7b. Toe III termination point; 7c. Toe IV termination point.

The flexor tendons on the plantar side of the toes are covered by a superficial fascia. These tendons overlay the proximal region of the tarsometatarsal bones, extending midway and delving into the adjacent tendon sheaths near the intertarsal joint’s distal extremity, as depicted in Figure 7A. Beneath these structures, the flexor tendons converge into a bundle at the tarsometatarsal bone, attaching distally to the respective phalanges of the second, third, and fourth toes. The metatarsophalangeal joints, enveloped by a retinaculum illustrated in Figure 7B, facilitate the redirection of the flexor tendon forces, functioning akin to a pulley. External to these is the tendon sheath encasing each tendon. Upon removal of the sheath, the tendons are revealed, and their intertendinous tissues are dissected using a scalpel as shown in Figure 7C, thereby isolating them for unobstructed examination. Each tendon originates from superior musculature, with its distal counterpart traversing the proximal tendon foramen. Subsequently, the terminating point of the tendon anchors to the articular cartilage within the articular fossa. This termination site aligns with the proximal extremity of the corresponding phalanx, primarily regulated by the tendon, thereby orchestrating the toe’s flexion process.

Figure 7E delineates the arrangement of tendons across toes II, III, and IV. In toe II, three distinct tendons are identified: the first tendon (2) aligns with the medial aspect of the articular fossa in the first phalanx, whereas the second tendon (3), characterized as a porous flexor tendon, associates with the articular fossa of the second phalanx. Similarly, toe III comprises three tendons, with the initial tendon (4)—another porous flexor variant—adjoining the articular fossa of the second phalanx. The subsequent tendon (5) is positioned at the articular fossa of the third phalanx, maintaining the porous flexor attribute. Contrastingly, toe IV presents a unique configuration. Its first tendon (6) resides on the lateral facet of the articular fossa within the inaugural phalanx. The second tendon (7) demonstrates a trifurcated arrangement, diverging into strands (7a–c) of varying lengths. These segments culminate at the articular fossae of the second, third, and fourth phalanges, respectively. Common to toes II, III, and IV is an extended flexor tendon (1), the third in the sequence for each toe. This tendon bifurcates distally, converging proximally at the metatarsophalangeal joints and extending distally to the respective phalanges. Specifically, it anchors at the distal articular heads of the second phalanx in toe II, the third phalanx in toe III, and the fourth phalanx in toe IV, as depicted in Figure 7D. Each bifurcation (1a–c) continues towards the nail. The precise termination points of these tendons were surgically severed and arranged for clear visualization in Figure 7F.

**Figure 8:**
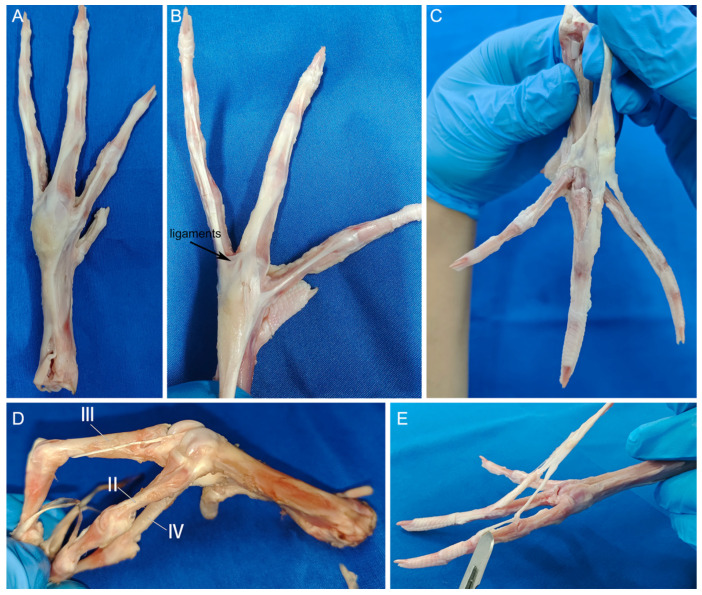
Extensor tendons, extensor tendon distribution (**A**). Ligament interconnecting the second and third toes (**B**). Extensor tendons post-proximal dissection (**C**). The first phalanx of the third toe’s tendon (**D**). Attachment relationships of extensor tendons (**E**). The Roman numerals II, III and IV represent the second, third and fourth toes of the mallard’s foot, respectively.

The dorsal side’s extensor tendons, shrouded in superficial fascia, present a contrasting configuration to their plantar counterparts (Figure 8A). These tendons attach to the metatarsophalangeal joints through fascia and eventually split into three separate bundles. They come together in a single structure near the distal end of the intertarsal joints (Figure 8C). These formations correspond to the second, third, and fourth toes (Figure 8E), each tendon subsequently bifurcating to connect to individual phalanges. This architecture facilitates synchronized, stable phalangeal motion upon muscular contraction. An anomalous feature of toe III is an extensor tendon originating laterally at the metatarsophalangeal joint and culminating at the first phalanx’s distal head (Figure 8D). 

The metatarsophalangeal joints employ finer, more delicate retinacula on their plantar (Figure 7B) and dorsal aspects (Figure 8A), assisting in redirecting tendon force and stabilizing tendon positioning. 

Ligaments are composed of robust connective tissue and bridge bones, aligning with tension direction and offering formidable tensile strength coupled with inherent elasticity. They reinforce joint structures and constrain skeletal motion. A notable ligament, positioned adjacent to the metatarsophalangeal joints, spans the third and fourth toes, anchoring at both extremities to the respective first phalanges (Figure 8B). This feature is conspicuously absent between the second and third toes. This discrepancy suggests that the interdigital ligaments between toes III and IV contribute to constraining their divergence angle.

#### 3.2.3. Skeleton

Table 2 presents detailed structural data, including the lengths of each phalanx, the diameters of the distal phalanges, and measurements at 1/4, 1/2, and 3/4 along the body of the phalanges, as well as the proximal sections.

##### Tarsometatarsus

**Figure 9:**
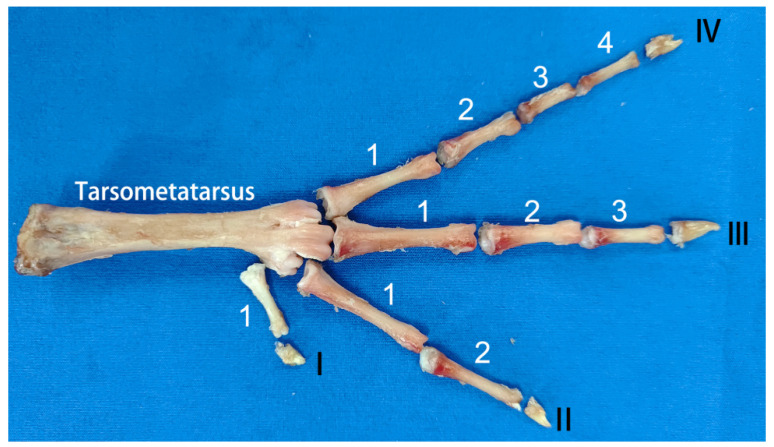
Anatomical arrangement of tarsometatarsals and phalanges. Toe I, II, III, and IV are composed of 1, 2, 3, and 4 phalanges, respectively.

The tarsometatarsus, characterized by its irregularity, exhibits a slender, anterior–posterior flattening along its longitudinal axis (Figure 9). These bones demonstrate notable thickness at both the proximal and distal ends, tapering significantly towards the center. The distal extremity divides into three articular heads, each dovetailing with the corresponding phalanges’ distal segments, forming what is recognized as the metatarsophalangeal joint. Conversely, the proximal end, shaped into an articular fossa, accommodates the tarsal bones’ distal segments, collectively comprising the intertarsal joint. Each metatarsophalangeal articulation is distinct; the leftmost head of the left foot’s distal tarsometatarsus and the first phalanx of toe IV constitute the IV metatarsophalangeal joint. The central articular head aligns with the first phalanx of toe III, creating the III metatarsophalangeal joint, whereas the rightmost connection involves the first phalanx of toe II, establishing the II metatarsophalangeal joint. An identical, symmetrical arrangement is observed in the right foot.

##### Phalanges

The phalanges, each irregularly shaped, span ten distinct segments, as depicted in Figure 9. The extremities of the four toes’ most distal phalanges culminate in robust, keratinized structures known as toenails. These proximal phalanges are conspicuously elongated and sizable, progressively diminishing in scale towards each toe’s tip. Each phalanx encompasses three primary components: a base, body, and head, with the base notably larger than the head. The configuration of these bones resembles a hinge, incorporating an articular fossa at the base and a prominent crest emerging along the articular fossa’s midsagittal zone. Additionally, a concave groove occupies the central region of the phalanx. This intricate alignment of the articular head and fossa facilitates the phalangeal joints’ uniaxial synovial functionality, restricting motion to elementary flexion and extension around the coronal axis.

The phalangeal joints are comprised of an articular surface, a joint capsule, and an articular cavity. The phalanges’ articular surfaces are covered in clear cartilage, and there is a collagen-filled synovial layer around them that helps the joints stay lubricated and reduces pressure and friction during movement. The joint capsule, a protective connective tissue sheath encompassing the joint, features an external layer of dense connective tissue providing joint stability and an internal layer of looser tissue responsible for the secretion of synovial fluid. This fluid not only lubricates the joint but also nourishes the cartilage. Adjacent phalanges are interconnected by both ligaments and inherent cartilaginous structures. The space enclosed by the articular cartilage and the joint capsule forms the joint cavity. The intricate morphology and robust ligamentous connections between the phalanges principally contribute to the foot’s structural integrity.

### 3.3. Texture Analyzer

**Figure 10:**
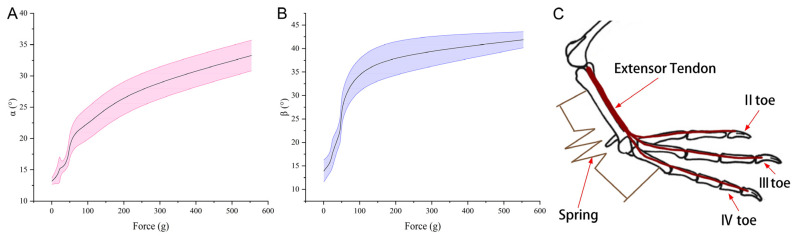
Correlation between the force dynamics of the tarsometatarsals and the webbing joint angles, specifically the α joint angle (**A**) situated between toes II and III and the β joint angle (**B**) located between toes III and IV. A simplified representation of the webbing’s mechanical deployment (**C**) is also provided, with the spring structure indicated in brown and the extensor tendon on the mallard foot’s dorsal side in red. The bone reference in (**C**) comes from Clifton et al. [6].

The interplay between force application and the resultant deflection of the toe webbing is illustrated in Figure 10. As the force correlates positively with the α and β joint angles, an escalation in these angles necessitates an augmented propulsive force, reaching a pinnacle at approximately 550 g.

## 4. Discussion

### 4.1. Rigid-Flexible Material Coupling Assembly and Biomechanical Functions

The material assembly characteristics of a mallard’s foot integrate the skeleton as the primary framework, establishing a supportive structure. Encased in the periosteum, the bones connect to tendons, facilitating bone movement. A protective sheath encompasses the tendons, safeguarding the tendon tissue while minimizing resistance to sliding and optimizing the transmission of muscular force through the tendon’s inherent elasticity. This elasticity accommodates internal forces, acting akin to a shock absorber during high-intensity activities by absorbing tension, thereby mitigating joint and tissue stress. The concerted function of muscles and tendons fortifies joints, ensuring dynamic stability and maintaining the joints’ correct positioning and motion range. Overlying the tendons, the skin forms a protective layer, incorporating a fat pad structure on the metatarsal side for shock absorption. This layer is further shielded by a stratum corneum with an irregular surface, safeguarding subcutaneous tissues and enhancing environmental adaptability.

Joint stabilization is achieved through abundant, robust ligamentous connections and reinforcements positioned dorsally, plantarly, medially, and laterally around the toes. The mallard’s gravitational force is conveyed to the tarsometatarsal bones through the intertarsal joints, and subsequently to the second, third, and fourth toes via the metatarsophalangeal joints. These joints exhibit bidirectional flexibility. Upon initial ground contact, the angle between the joint and the dorsal side approaches 180 degrees, progressively decreasing until ground departure, at which point it reverses direction, ultimately bending toward the plantar side during the swing phase. The joints’ morphological and structural features, along with the interosseous ligaments, primarily contribute to maintaining joint stability. Ligamentous tendons within and surrounding the metatarsophalangeal joints are crucial for effective force transmission.

Upon initial ground contact, the mallard foot first makes contact through the toe tips, swiftly followed by the entire palm’s descent. The elastic fat pad on the metatarsal aspect of the foot assumes a critical cushioning function, absorbing shock. This feature bears resemblance to the adaptive structures found in the footpads of elephants [7], ostriches [5,8], shepherd dogs [9], and goat hooves [11]. The skin and adipose tissue are instrumental in safeguarding the skeletal and tendon systems. During joint articulations, the skin exhibits heightened activity, corresponding with a substantial elastic modulus. In contrast, the fascia follows suit, and the toe pads, tasked primarily with bearing compressive rather than tensile loads, present the lowest elastic modulus. Tendons, necessitated to endure greater tension, are inherently slender and exhibit a higher modulus of elasticity. Consequently, the skin, characterized by a superior elastic modulus, serves a protective function, enveloping other components like toe pads and tendons, which possess a lower elastic modulus. Concurrently, the pronounced adipose layers adjacent to the bones shield the skeletal structures. Mallard skeletons, demonstrating higher aspect ratios, adopt an irregular configuration, manifesting numerically as increased thickness at the extremities and reduced thickness medially. This nuanced design enhances the range of motion, foot surface area, and flexibility while preserving functional integrity. The asymmetrical architecture imparts augmented strength to the joint heads and fossae, bolstering joint stability. This strategic reduction in bone mass retains structural robustness without compromising weight efficiency, a structural paradigm also observable in the ostrich foot [12].

### 4.2. Osteomuscular Motor System and Biomechanical Function

The intricate tendon system within the mallard’s hindlimb underpins its locomotion, furnishing both strength for ambulatory activities and traction for various bodily components through elongated, lightweight tendons. Muscle distribution predominantly features the femoral and tibiotarsal regions, with comparatively minimal musculature extending below the tarsal joint. Locomotion hinges primarily on tendon-driven bone movement, a mechanism that minimizes the weight and physical bulk associated with the more dynamic tarsometatarsus and feet [1]. This streamlined structure equips the mallard with sustained functional capacity during walking and swimming activities.

**Figure 11:**
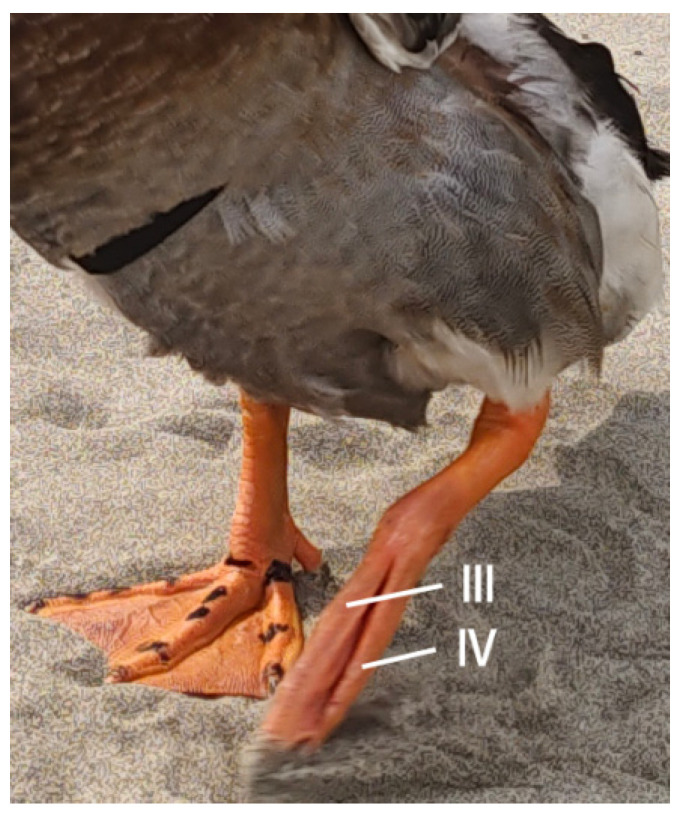
Mallard in left-foot flexed stance. The Roman numerals III and IV represent the third and fourth toes of the mallard’s foot, respectively.

When walking on land or running on solid ground, the dorsal extensor tendons and metatarsal flexor tendons work together to make the complex processes of digital flexion and extension happen. The flexor tendon, as the main tendon for toe flexion, plays an important role in the storage and release of elastic strain energy, which is similar to the ability of the horse long flexor tendon to store and utilize a considerable amount of elastic strain energy while standing [14]. Notably, the dorsal region houses fewer tendons, both in quantity and size, than its plantar counterpart (refer to Figure 7 and Figure 8). The mallard’s foot extensor tendon splits into three strands at the tarsometatarsals. Each strand connects to toes II, III, and IV, coordinating their extension movements. This anatomical arrangement accounts for the uniform alteration in the vertical elevation of the mallard’s toes upon lift-off [17]. Specifically, the extensor tendon originating at the lateral facet of the third toe’s metatarsophalangeal joint and culminating at the distal articulatory crest of this toe’s primary phalanx segment (illustrated in Figure 8D) imposes a restriction on the toe’s plantar flexion angle. When this angle’s upper limit is reached, this tendon is at its most tense. This shows why toe III’s flexion angle is less than its metatarsophalangeal joint during the swing phase of the foot. This special arrangement of tendons, which is not found in the other toes, makes the flexion angle on the metatarsal side more noticeable. This places toe III in front of toes II and IV during the aerial phase of the swing (Figure 11).

Further biomechanical insights were gleaned from testing the tarsometatarsal-driven webbing expansion (shown in Figure 10). Here, the webbing’s aperture angle correlated positively with the downward pressure exerted on the tarsometatarsal. The force’s intensity mirrored the widening angle of the webbing, underscoring the role of the metatarsophalangeal joint reduction in facilitating webbing extension. Additionally, the mallard’s consistent webbing angle during ground contact implicates the tarsometatarsal’s passive engagement. This organic architecture bestows energy efficiency upon the mallard’s ground contact, negating the need for supplementary muscular exertion and thereby enhancing walking efficacy.

The tarsometatarsal bone rotates anteriorly during webbing deployment, which also reduces the angle between the metatarsophalangeal joint and the toes. This movement originates from an extensor tendon contraction, simultaneously extending the flexor tendon, thereby exerting a distal pull on the muscle and generating resistance. Simplistically, the tarsometatarsal bone’s rotation directly influences the toe webbing’s aperture modulation, with the metatarsal-side flexor tendons of the tarsometatarsal bone analogized as springs. These connective components link one extremity to the tarsometatarsal bone and the opposite to toes II and IV. This rotational action extends the metaphorical springs along the metatarsal aspect, guiding toes II and IV outward. Within this model, the spring embodies a nonlinear passive structure (refer to Figure 10C) (Harrison et al., 2010) [14].

Nonetheless, certain constraints merit acknowledgment. The inherent narrowness of extensor tendons and the fascia’s robust adhesive characteristics posed challenges for detailed anatomical scrutiny. Experimental confines and temporal restrictions precluded an exhaustive exploration of extensor tendon intersections, an aspect warranting further attention in ensuing dissections of the mallard foot.

## 5. Biomimetic Foot Design

### 5.1. Structure Composition

We have conceptualized a biomimetic foot in light of the mallard foot’s intricate anatomical structure and earlier kinematic studies [17,18]. This construct amalgamates a biomimetic webbing articulation mechanism, a segmental multi-toe elevation system, and synthetic webbing. The articulatory ensemble comprises a biomimetic tarsometatarsal component, torsion springs, a coiled column, a spiral slider, reciprocal blocks, and bearing supports. Concurrently, the multi-toe lift-off apparatus encompasses the second, third, and fourth digits. Mimicking the mallard’s skeletal configuration, the mechanical foot’s foundational structure replicates the precise bone arrangement, both in position and count. These synthetic bones are encased in Supplementary Materials emulating skin and internal tissues. These elastic substances are strategically positioned on the digits, with interdigital materials simulating natural webbing. The foot’s heel region employs similarly pliant materials, designed to replicate the cushioning adipose pads found on the mallard’s sole.

The biomimetic foot adopts the proportional dimensions of a mallard’s foot. Based on the measured parameters of the toe bones, we mimic these proportions to design the sizes of the individual toe bones in the biomimetic foot. The material for the toe bones can be high-strength steel or other rigid materials, whereas the material for the biomimetic webbed foot is high-performance TPU material, renowned for its exceptional flexibility. The combination of rigid toe bones and the pliable webbed foot pad forms a rigid-flex coupling assembly. The biomimetic foot possesses variable surface area functionality, primarily suited for soft ground applications, offering excellent resistance to sinking. At present, the biomimetic foot has not fully applied the tendon-bone motion model. In the future, we will continue to research tendon-driven biomimetic feet.

### 5.2. Engineering Bionic Principle

**Figure 12:**
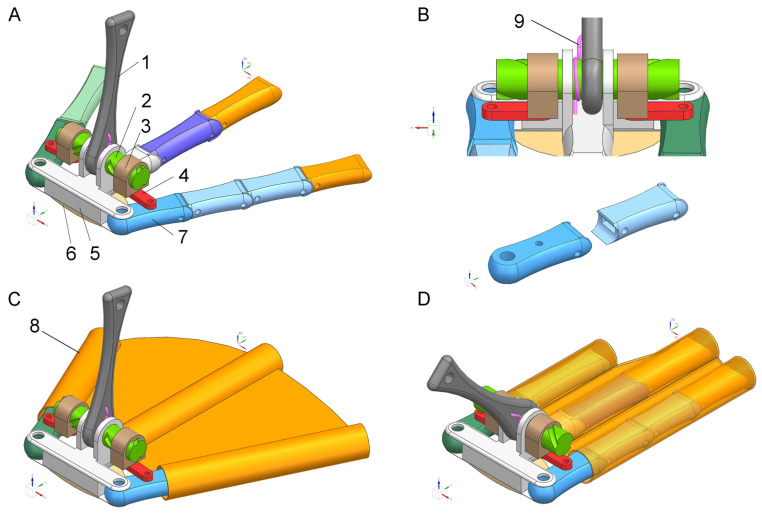
Bionic foot design. Illustration showcasing the webbing’s expanded state (**A**) and detailed view (**B**) pertaining to the bionic foot, accompanied by representations of the webbing in both expanded (**C**) and retracted (**D**) conditions post-attachment. Components: 1. bionic tarsometatarsal bone; 2. helical post; 3. helical slider; 4. push-pull block; 5. base; 6. bionic foot pad; 7. bionic phalanges; 8. bionic webbing; 9. torsion spring. The red, green and blue arrows represent the X, Y and Z axes, respectively.

As depicted in Figure 12, the design emulates the mallard foot’s functionality, particularly the opening of the webbing via the second and fourth toes prior to ground contact and its closure post-lift-off. This mechanism involves the outward splaying of the second and fourth toes, initiated by the forward rotation of the bionic tarsometatarsal bone during ground contact. This action triggers the expansion of the bionic webbing, augmenting the contact area and enhancing the bionic foot’s sinkage resistance. At the same time that lift-off starts, the tarsometatarsal bones start to recoil, controlled by torsion springs. This causes the bionic second and fourth toes to close and the webbing to retract, which reduces aerodynamic drag while the bionic foot moves.

To emulate the sequential ground-release motion of a mallard’s three toes, the bionic foot incorporates multi-segmented replica toes. These artificial phalanges interconnect through a synergistic mechanism involving simulated joint heads and fossae. Joint restrictions are achieved via a carriage assembly, whereas tension spring preloading simulates the authentic joint’s tensile framework. This design ensures that the bionic foot achieves enhanced relaxation upon ground release and increased flexibility upon ground contact without necessitating an auxiliary control system.

In order to mimic the energy-saving effect observed in the mallard’s tendons, torsion springs are integrated into the metatarsophalangeal joints. This feature passively accumulates energy during forward motion and subsequently dispenses this stored energy upon ground release, facilitating effortless hindfoot lift-off. Additionally, biased tension springs are installed within the phalangeal joints, assisting the distal phalanges in ground detachment and reversion to their initial stance. This system also serves to dampen and diminish the vibrational impact experienced by the bionic foot upon touching the ground.

### 5.3. Working Process

Upon ground contact, the mallard foot initiates contact via the foot’s tip, subsequently followed by full palm grounding. The action of the second and fourth toes orchestrates the expansion of the interdigital webbing. The third toe, being the most extended, makes initial contact, guiding the rapid grounding of the foot’s palm through a pivot around the third toe’s tip. Concurrently, a forward rotation of the tarsometatarsal bone around the metatarsophalangeal joints ensues during this touchdown phase, resulting in a reduced joint angle. As lift-off commences, the elevation of the tarsal bones triggers the retraction of the hindfoot, progressively increasing the metatarsophalangeal joint angle. This sequence continues with the phalanges sequentially disengaging from the ground, commencing with the proximal and concluding with the distal toes. The lift-off phase culminates with this full retraction. During the swing phase, the second and fourth toes instigate a gradual closure, pulling the webbing inwards and extending the toes to facilitate webbing expansion in preparation for the subsequent stride cycle, thereby completing one full stride sequence.

In parallel, the bionic foot, upon ground contact, leads with the tip due to its forward center of gravity and the inherent angle of the bionic metatarsophalangeal joints. The full grounding of the palm follows this initial contact quickly. This transition is facilitated through a forward rotation of the bionic tarsometatarsal bone, inducing a clockwise motion of the helical column. This rotation translates to the helical slider instigating its motion, which, in turn, propels the push-pull block. Consequently, the simulated second and fourth toes undergo simultaneous extension, driving the expansion of the bionic webbing. This sequence augments the contact surface, mitigating sinking by distributing the load over a broader base.

During lift-off, the bionic foot initiates its ascent with the torsion spring at the tarsometatarsal bone reverting to its original position, thereby releasing stored energy. This action prompts the proximal phalanx to rotate around the first phalanx due to the metatarsophalangeal joint’s torsion spring, initiating ground detachment. Upon reaching the toe joints’ maximal rotational angle, the phalanx finalizes its lift-off sequence, commencing with the first phalangeal of the second, third, and fourth toes. After that, the torsion spring causes the remaining distal phalanges to separate one at a time until the last distal phalanx moves off the ground. The bionic foot’s lift-off phase is now complete. This progression is similar to the phalanges’ sequential detachment and reduces both adhesive ground forces and environmental perturbations.

When the bionic foot swings, the torsion spring at the tarsometatarsal bone re-engages, releasing any stored energy and starting the backward rotation of the tarsometatarsal bone. This motion induces a counterclockwise rotation in the helical column, propelling the helical slider and reinstating the push-pull block to its initial stance. Consequently, the bionic second and fourth toes commence closure, and the bionic webbing capitalizes on its elastic potential energy to facilitate its contraction. This process reduces aerial forward movement resistance, marking the completion of a singular stride cycle for the bionic foot.

Webbing dynamics in the bionic foot are controlled by the bionic tarsometatarsal bone’s forward rotation. The bionic metatarsophalangeal joint’s torsion spring controls the bone’s retraction. This passive modulation of the webbing confers dual advantages: the obviation of a control system and a reduction in energy expenditure. Through the rigid-flexible synergy between the toe and webbing, the bionic foot adeptly maximizes ground contact area, enhancing friction and sinkage resistance during touchdown. Conversely, it minimizes this area during lift-off to reduce aerial resistance and ground-touch vibration.

## 6. Conclusions

The material and structural assembly of the mallard foot showcase distinct characteristics: the skeleton forms the primary framework, establishing a supportive structure, whereas the tarsometatarsal bone exhibits attributes of a lightweight yet high-strength functional structure. Tendons, affixed above the bones, facilitate bone movement and are enveloped by tendon sheaths that safeguard the tendon tissues and minimize the friction encountered during tendon sliding. Encasing these components, the skin envelops both tendons and bones, supplemented by fat pads located on the plantar aspect of the foot to provide cushioning.

In terms of the osteomotor system, the mallard foot distinguishes itself primarily through its flexor tendons, which are more robust and distinctly segmented compared to the slimmer extensor tendons that converge into a single bundle at the proximal tarsometatarsal bones. During ambulatory activities, such as walking or grounded running, there is a harmonious interaction between the extensor tendon, located dorsally, and the flexor tendon, situated plantarly, orchestrating the toes’ flexion and extension movements.

Furthermore, the opening angles of the webbing are in direct proportion to the pressure exerted on the tarsometatarsal. This observation has led to the proposal of a biomechanical model. This study delved into the intricate bio-structural properties and material assembly of the toe and webbing, as well as the kinematic system encompassing the bones and muscles within the mallard’s foot. These insights contribute valuable data for future anatomical explorations into the feet of webbed birds but also inform the creation of a bionic foot prototype. On the basis of the mallard foot study, a bionic foot was designed, which provides a reference and a lesson for the future design of bionic feet. 

## Figures and Tables

**Table 1:** Presents a detailed breakdown of the CT-analyzed parameters associated with the tarsometatarsal bones.

Description	Abbreviation	Unit	Value
Bone mineral density	BMD	g/cm^3^	0.3025592
Tissue volume	TV	mm^3^	200.1423233
Bone volume	BV	mm^3^	58.51021879
Percent bone volume	BV/TV	%	29.23430578
Tissue surface	TS	mm^2^	349.4054717
Bone surface	BS	mm^2^	925.5858307
Intersection surface	i.S	mm^2^	56.67429698
Bone surface/volume ratio	BS/BV	1/mm	15.81921671
Bone surface density	BS/TV	1/mm	4.62463818
Trabecular pattern factor	Tb.Pf	1/mm	2.30657951
Structure model index	SMI	-	0.75189734
Trabecular thickness	Tb.Th	mm	0.21542645
Trabecular number	Tb.N	1/mm	1.28619386
Trabecular separation	Tb.Sp	mm	0.80514495

**Table 2:** Metrics pertaining to the tarsometatarsal bones and individual phalanges in mallards.

Measurement Objects	Distal		1/4		1/2		3/4		Proximal		Length/mm
	Max	Min	Max	Min	Max	Min	Max	Min	Max	Min	
Toe Ⅰ, 1st phalanx	3.93	2.85	3.43	2.77	3.71	2.87	4.21	3.79	5.12	4.88	14.33
Toe Ⅱ, 1st phalanx	4.83	3.55	3.53	2.81	3.69	3.35	5.35	4.49	6.58	6.27	30.53
Toe Ⅱ, 2nd phalanx	3.78	2.4	2.86	2.51	2.92	2.43	3.55	3.33	5.17	4.74	23.67
Toe Ⅲ, 1st phalanx	5.77	4.99	4.17	4.07	4.66	4.26	6.87	5.33	9.27	8.89	30.87
Toe Ⅲ, 2nd phalanx	4.43	3.99	3.38	3.15	3.81	3.62	5.19	4.37	6.36	6.23	21.88
Toe Ⅲ, 3rd phalanx	3.81	2.95	3.23	2.54	3.21	2.80	3.37	4.18	5.49	4.81	18.42
Toe Ⅳ, 1st phalanx	5.73	4.74	3.35	3.18	3.75	3.58	5.21	5.05	7.18	6.67	26.00
Toe Ⅳ, 2nd phalanx	4.24	3.72	3.06	2.92	3.94	3.17	4.63	4.24	5.63	5.57	17.68
Toe Ⅳ, 3rd phalanx	3.35	3.25	2.64	2.40	2.79	2.66	3.45	3.10	4.77	4.40	13.41
Toe Ⅳ, 4th phalanx	3.36	1.95	2.44	1.92	2.33	2.10	2.95	2.24	4.44	4.13	15.32
tarsometatarsal	12.94	9.91	7.66	5.25	7.38	6.26	8.44	6.38	15.72	14.33	66.24

## Data Availability

The data presented in this study are available on request from the corresponding author. The data are not publicly available due to our laboratory privacy data protection.

## Supplementary Materials

The following supporting information can be downloaded at: https://www.mdpi.com/article/10.3390/biomimetics8080592/s1, Video S1: Extensor Tendons.
